# Molecular Properties of Bare and Microhydrated Vitamin B5–Calcium Complexes

**DOI:** 10.3390/ijms22020692

**Published:** 2021-01-12

**Authors:** Davide Corinti, Barbara Chiavarino, Debora Scuderi, Caterina Fraschetti, Antonello Filippi, Simonetta Fornarini, Maria Elisa Crestoni

**Affiliations:** 1 Dipartimento di Chimica e Tecnologie del Farmaco, Università di Roma “La Sapienza”, Piazzale Aldo Moro, 5, I-00185 Roma, Italy; davide.corinti@uniroma1.it (D.C.); barbara.chiavarino@uniroma1.it (B.C.); caterina.fraschetti@uniroma1.it (C.F.); antonello.filippi@uniroma1.it (A.F.); simonetta.fornarini@uniroma1.it (S.F.); 2 Institut de Chimie Physique (UMR8000), CNRS, Université Paris-Saclay, 91405 Orsay, France; debora.scuderi@universite-paris-saclay.fr

**Keywords:** pantothenate, vitamin B5, calcium, complexation, IRMPD spectroscopy, quantum chemistry calculations, molecular structure

## Abstract

Pantothenic acid, also called vitamin B5, is an essential nutrient involved in several metabolic pathways. It shows a characteristic preference for interacting with Ca(II) ions, which are abundant in the extracellular media and act as secondary mediators in the activation of numerous biological functions. The bare deprotonated form of pantothenic acid, [panto-H]^−^, its complex with Ca(II) ion, [Ca(panto-H)]^+^, and singly charged micro-hydrated calcium pantothenate [Ca(panto-H)(H_2_O)]^+^ adduct have been obtained in the gas phase by electrospray ionization and assayed by mass spectrometry and IR multiple photon dissociation spectroscopy in the fingerprint spectral range. Quantum chemical calculations at the B3LYP(-D3) and MP2 levels of theory were performed to simulate geometries, thermochemical data, and linear absorption spectra of low-lying isomers, allowing us to assign the experimental absorptions to particular structural motifs. Pantothenate was found to exist in the gas phase as a single isomeric form showing deprotonation on the carboxylic moiety. On the contrary, free and monohydrated calcium complexes of deprotonated pantothenic acid both present at least two isomers participating in the gas-phase population, sharing the deprotonation of pantothenate on the carboxylic group and either a fourfold or fivefold coordination with calcium, thus justifying the strong affinity of pantothenate for the metal.

## 1. Introduction

**Scheme 1:**
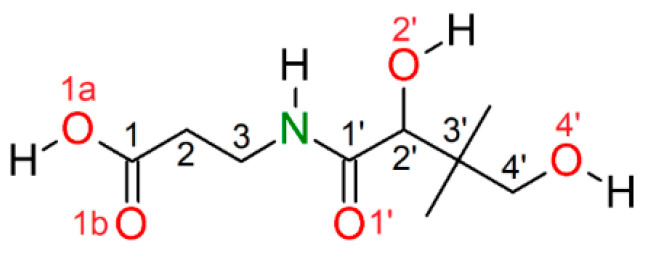
Schematic representation of (R)-pantothenic acid. Annotation for atom numbering is reported.

(R)-pantothenic acid (Scheme 1), also known as vitamin B5, is a water-soluble nutrient that is essential for growth, reproduction, and tissue restoration of humans and animals. As a structural component of coenzyme A (CoA) and of the acyl carrier protein, vitamin B5 (vit B5) recovers CoA levels, thus increasing mitochondrial activity [1]. Hence, coenzymes containing pantothenic acid are involved in biological functions that mainly affect energy production and lipid metabolism [2]. Interestingly, some pantothenic acid amides have recently revealed effective antimicrobial activity by targeting the CoA biosynthetic pathway [3].

Clinical deficiency of vit B5 is uncommon, because it is widely distributed in the daily diet and is supplied by gut microbiota, similarly to other B vitamins [4]. However, a reduced intake may arise in individuals with augmented requests or result from inapt food processing procedures and storage. Indeed, the pH-sensitive peptide bond between β-alanine and pantoic acid in vit B5 allows its release only by enzymatic action.

The deficit of pantothenic acid may adversely alter the immune system response as well as reduce cortisol and acetylcholine production, eventually leading to extensive pro-inflammatory effects, myalgia, and depression.

Lately, the expression of vanin-1, a vascular non-inflammatory enzyme that breaks down pantetheine in cysteamine and pantothenic acid, has been discovered to regulate cell migration, redox balance, and a number of metabolic pathways. Its physiological roles, strictly associated to coenzyme A metabolism, uncover networked actions in different organs, posing it as either a therapeutic target or a valuable candidate biomarker of various disease progressions [5,6].

In addition to its widespread use as a nutraceutical in multivitamin supplements, vit B5 and derivatives (e.g., pantothenol, pantethine) find recognized application in skin-care products, including cosmetics and drug-loaded wound dressing, which is acknowledged to significantly enhance the healing process [7,8]. Indeed, as a result of the proliferation and migration of dermal fibroblasts, pantothenic acid does promote skin regeneration and facilitate a successful healing of burn and surgical wounds [9].

The continuous attention for pantothenic acid uses has defined the selection of advanced extraction, separation, and determination procedures that can ensure its accurate analysis in natural and industrial preparations. In addition to microbiological assays, chromatography coupled with ultraviolet or fluorescence detection, and radioimmunoassay, highly sensitive HPLC electrospray mass spectrometry (MS) has recently been applied as reliable method for B-vitamins determination [10,11,12,13].

In complex matrices such as food, the excellent sensitivity and accuracy of high-resolution MS allows probing low-abundance, small-molecule metabolites, providing reliable results as required in current nutrition science [14]. However, MS suffers from limited structural information that prevents a definitive molecular characterization and needs the coupling with an orthogonal technique such as vibrational spectroscopy [15,16,17,18,19,20,21].

In recent years, infrared multiple photon dissociation (IRMPD) spectroscopy has been widely applied, providing ample and specific structural information on the chemical arrangement of a variety of (bio)molecular ions and metal complexes in the gas phase without bulk solvent and counter ion effects [22,23,24,25,26,27,28,29,30]. This vibrational spectroscopy relies on the sequential, resonant absorption of multiple IR photons followed by a fast intramolecular vibrational redistribution (IVR) that eventually induces structure-dependent photofragmentation revealed by mass spectrometry [15]. IRMPD has been exploited to investigate metal [31,32,33,34,35] and halide [36] ion binding patterns, the (de)protonation of (bio)molecular ions [23,37,38,39], fragmentation routes [40], post-translational modifications of amino acids and peptides [21], isomeric discrimination [41,42,43,44], and, more recently, biomarker discovery for medical diagnostics [45,46,47].

The unequivocal differentiation of enantiomeric N-acetylhexosamines has been also demonstrated, confirming the potential of IRMPD to uncover biomarkers in clinical screening of inborn errors of metabolism [48].

An approach based on electrospray ionization (ESI) and IRMPD spectroscopy as biophysical tools backed by quantum chemical calculations is reported here to expand our information on vitamin B5 at the molecular level, shedding light on the energetics, geometric determinants and photofragmentation behavior of pantothenic acid upon binding with the naturally occurring Ca(II) ion. The perspective is to relate structural features to the biochemical functions of this essential micronutrient. Overall, the role of Ca(II) binding properties is of great significance in various biological systems, such as information transmission and amplification, memory, and triggering muscle contraction [49].

In the last decade, a large effort has been devoted to the structural characterization of metal ion complexes with several biomolecules in the gas phase by using IRMPD in the highly diagnostic mid-infrared range and theoretical calculations, concurring to distinguish multidentate binding patterns. In the presence of doubly charged metal ions, the coordination of amino acids and small peptides may occur at carboxylic acid, the amine group, the amide backbone, or the side chain thiol site, leading to singly charged metal complexes [50]. While weakly binding alkali and alkaline earth metal ions (such as Ca(II)) are found to prefer charge-solvated (CS) adducts interacting with oxygen atoms as Lewis-basic chelation sites, as in the calcium transporter protein calmodulin [51], and may keep a water ligand, strongly ligated transition metal ions bind to deprotonated amide nitrogens via iminol tautomerization. Examples in this class are Ni(II), which is known to bind with human serum albumin at the N-terminus [52,53,54], and Cu(II) with the prion protein [55,56]. Consequently, different coordination schemes clarified in gaseous complexes are strictly related to the biological roles of metal ions in the biological systems.

This paper follows from a preliminary study [57] on pantothenic acid by focusing on the singly charged complex consisting of the adduct between Ca(II) and the conjugate base of pantothenic acid.

In previous work, we have reported that the protonation of pantothenic acid favors the formation of a couple of conformers protonated at the amide carbonyl oxygen, which was found to be thermodynamically the most basic site of (panto + H)^+^ [57].

The focus here is to elucidate the binding sites either in a solvent-free form, [Ca(panto-H)]^+^, or microsolvated by one water molecule in [Ca(panto-H)(H_2_O)]^+^. The native conjugate base of pantothenic acid, [panto-H]^−^, has been also examined to provide a useful guide to interpret relevant spectroscopic features.

## 2. Results and Discussion

### 2.1. Mass Spectra and Fragmentation Channels

Electrospray ionization (ESI) in the positive ion mode of an aqueous methanol solution of calcium pantothenate yields an intense signal at *m*/*z* 258 consistent with the formation of the complex between calcium and deprotonated pantothenic acid, [Ca(panto-H)]^+^ (see Figure S1a in the Supplementary Materials). The presence of an open coordination sphere in the bare complex [Ca(panto-H)]^+^ justifies the presence of peaks attributed to a subsequent [Ca(panto-H)]^+^ water adduct. An even more intense signal at *m*/*z* 276 is ascribed to the singly hydrated [Ca(panto-H)(H_2_O)]^+^ ions.

When operating by ESI in the negative ion mode, the above solution, added with 2% ammonia to assist deprotonation, delivers the conjugate base of pantothenic acid, [panto-H]^−^, as shown in the mass spectrum reported in Figure S1b, where any hydrated complex is hardly detected.

The mass-selected species have been interrogated either by the IR absorption of multiple resonant photons or in a collision event by low-energy collision induced dissociation (CID). Comparable fragmentation patterns are observed for all sampled ions inasmuch both methods promote a slow heating process, which follows dissociation along the lowest energy channel [15].

The activation of [Ca(panto-H)(H_2_O)]^+^ proceeds by the exclusive elimination of water. Figure S2 as shown in the sample mass spectra of [Ca(panto-H)(H_2_O)]^+^ recorded either prior to (top trace) and after (bottom trace) irradiation with Centre Laser Infrarouge d′Orsay (CLIO) light tuned on resonance at 1460 cm^−1^.

The photofragmentation of electrosprayed [Ca(panto-H)]^+^ ion proceeds along competitive routes, affording the primary ions at *m*/*z* 240, by the removal of water, and at *m*/*z* 200, by loss of a 58 Da unit, [2H,2C,2O]. In turn, the former fragment at *m*/*z* 240 undergoes a consecutive elimination of a 30 Da unit, [2H,C,O]. Mass spectra recorded before and after exposure of [Ca(panto-H)]^+^ ions to IR free electron laser (FEL) on resonance at 1630 cm^−1^ are presented in Figure S3.

In order to compare the influence of metalation/deprotonation on the energetic, structural, and spectroscopic features of pantothenic acid, its conjugate base, [panto-H]^−^, has been probed in parallel by IRMPD in the same region explored for the above calcium complexes, leading to deprotonated β-alanine at *m*/*z* 88 and to a minor product ion at 146 by loss of a 72 Da unit, [2C,3O]/[CO_2_,CO]. Figure S4 compares the mass spectra obtained either without or with exposure of [panto-H]^−^ to CLIO light at 1510 cm^−1^.

### 2.2. IRMPD Spectra

**Figure 1:**
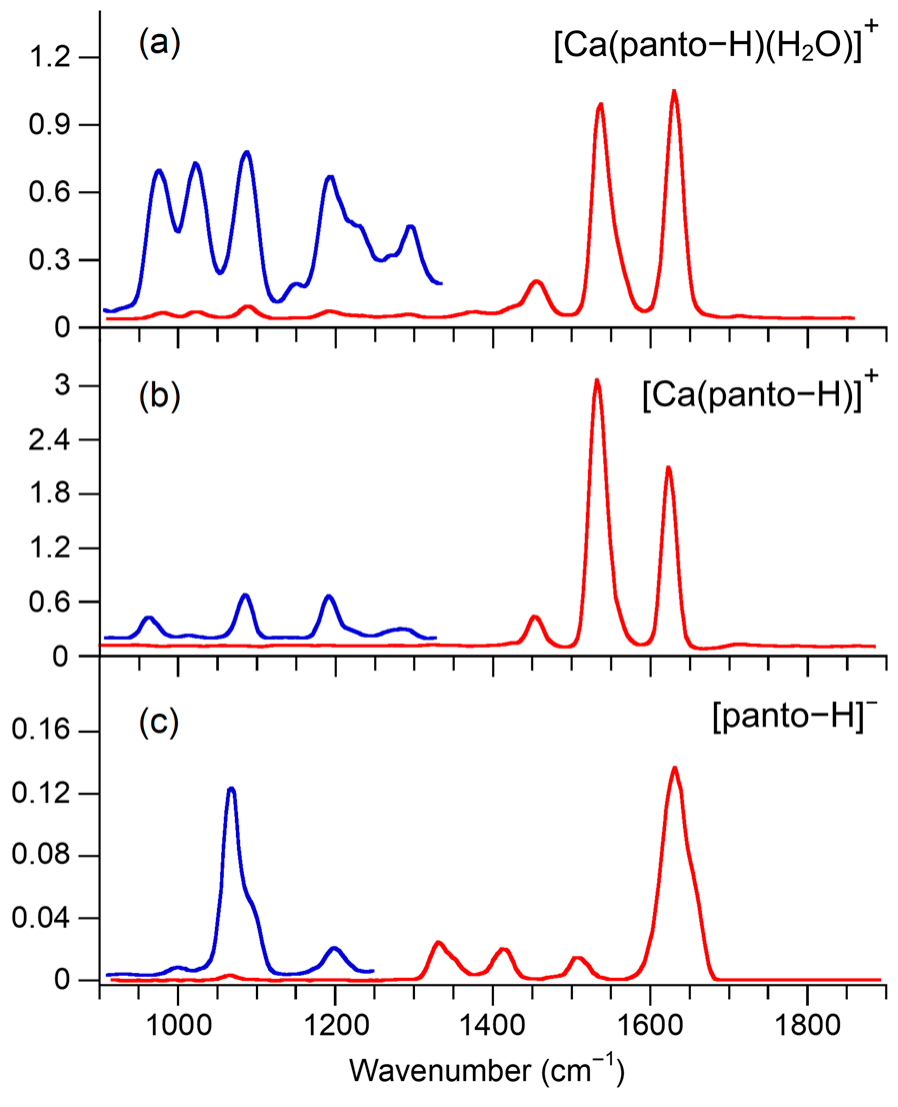
Experimental infrared multiple photon dissociation (IRMPD) spectra of: (**c**) deprotonated pantothenic acid [panto-H]^−^ when the ions are submitted either to 500 ms of free electron laser (FEL) light (blue profile) or to 120 ms using one attenuator (red profile); (**b**) pantothenate calcium complex [Ca(panto-H)]^+^ irradiated with either 1 s of FEL light (blue profile) or with 500 ms using one attenuator (red profile); (**a**) and its monohydrated form [Ca(panto-H)(H_2_O)]^+^ with 500 ms of FEL light and one attenuator (blue profile) or with 250 ms and three attenuators (red profile).

The structural features of ESI-generated [panto-H]^−^, [Ca(panto-H)]^+^ and [Ca(panto-H)(H_2_O)]^+^ ions have been investigated as bare species free of any external perturbation by IRMPD spectroscopy in the highly structurally revealing fingerprint range (900–1900 cm^−1^), by means of recording a wavenumber-dependent fragmentation process (Figure 1). The IRMPD process activates the same fragmentation paths in the whole inspected IR range, leading to the product ions described in Section 2.1. Where multiple product ions are formed, their ratio is constant throughout the explored IR range, indicating that the same ion or ion mixture is in fact sampled.

In this region, diagnostic absorptions by the peptide backbone, including the Amide I (C = O stretch) and Amide II (NH bend) modes, may be exposed [58].

The IRMPD spectrum of [panto-H]^−^ is dominated by a broad and intense absorption around 1632 cm^−1^. Distinct peaks are also observed at 1514, 1415, and 1334 cm^−1^, and some weaker absorbances appear below 1300 cm^−1^.

Interestingly, the interaction of pantothenate ion with calcium generates a spectroscopic signature around 1535 cm^−1^. In fact, both bare [Ca(panto-H)]^+^ and singly hydrated [Ca(panto-H)(H_2_O)]^+^ adducts exhibit new bands at 1530 and 1537 cm^−1^, respectively, of comparable intensity as the one around 1630 cm^−1^. The IRMPD spectra of the two calcium complexes, [Ca(panto-H)]^+^ and [Ca(panto-H)(H_2_O)]^+^, are indeed very similar and present the same major vibrational features, suggesting a shared geometric arrangement. Eventually, the interaction with water allowed obtaining a better characterization of the vibrations below 1500 cm^−1^. This effect can be attributed to the ease of the water cleavage that enables the observation of vibrational modes with lower intensity. Thus, water is also acting as a tagging molecule, analogously to what occurs in cryogenic ion traps where non-covalent complexes of the species of interests with rare gas atoms, e.g., He or Ar, are formed and submitted to IR ion spectroscopy [59,60].

Overall, calcium binding confers an evident mark to the vibrational features of the pantothenate ion.

### 2.3. Computed Structures and Spectral Assignments of [Panto-H]^−^

**Figure 2:**
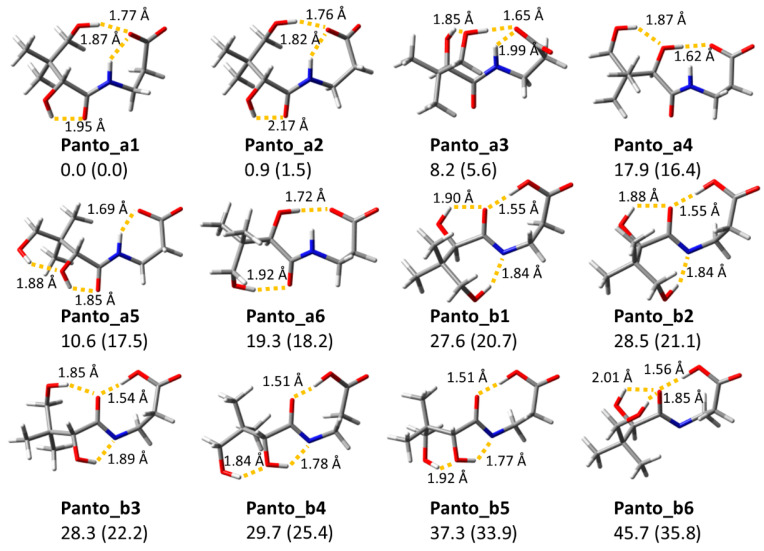
Structures of [panto-H]^−^ optimized at the B3LYP/6-311++G(d,p) level. Hydrogen bonds are reported with dashed lines together with their length (Å). Relative free energies at 298 K in kJ mol^−1^ are computed at the B3LYP and MP2 level (in parentheses).

To aid in the interpretation of the IRMPD spectrum, which mainly reflects the absorption of the first resonant IR photon, the conformational space of the conjugate base of deprotonated pantothenic acid, [panto-H]^−^, has been widely explored, starting from trial isomers envisaged on the basis of the deprotonation site, at either the carboxylic group or amide backbone. The optimized structures are reported in Figure 2 together with relative energies obtained by both DFT and MP2 calculations (details are described in Section 3.3), while the IR spectra of low-energy structures featuring different hydrogen bonding motifs are shown in Figures S5 and S6. For any given constitutional isomer, several low-lying rotamers resulting from rotation about single bonds in the flexible skeleton of [panto-H]^−^ have been identified. All the relevant thermodynamic data, including the relative enthalpy (ΔH°_rel_) and free energy (ΔG°_rel_) values at 298 K (kJ mol^−1^), determined at the B3LYP(-D3)/6-311++G(d,p) and single point MP2(full)/B3LYP/6-311++G(d,p), are summarized in Table S1. Henceforth, the relative free energy values will be provided at the B3LYP (in plain text) and MP2 (in parentheses) levels.

The energy ordering of the conformers, all characterized by charge-solvated (CS)/no salt-bridge zwitterionic structures, is very similar at both levels of theory. The carboxylic acid is the lowest energy deprotonation site, and an ensuing family of six rotamers (**Panto_a1–6**), corresponding to the most favorable arrangements of [panto-H]^−^ have been identified within a 19.3 (18.2) kJ mol^−1^ Gibbs free energy range (Figure 2).

The two lowest lying structures resulting from deprotonation at either carboxylic (**Panto_a1**) or amide (**Panto_b1**) functionality show the negative charge delocalized on either the carboxylate or amide group, respectively (Figure 2).

The global minimum **Panto_a1** adopts a rather folded arrangement stabilized by three intramolecular hydrogen bonds that encompass a bifurcated motif of the negatively charged carboxylate acceptor with both the primary hydroxyl unit (r_O1…HO4′_ = 1.771 Å) and the amide hydrogen (r_O1…HN_ = 1.869 Å) donors, and the interaction between the carbonyl oxygen O1′ with the secondary alcoholic center (r_O1′…HO2′_ = 1.955 Å). The six low-lying rotamers (**Panto_a1–6**) shown in Figure 2 include conformations very much alike the global minimum (in **Panto_a2**), which are more crumpled (in **Panto_a3**) or elongated (in **Panto_a4–6**). It turns out that the lack of an O1^−^…HO4′ hydrogen-bonding interaction causes a major destabilization in the latter geometries.

When deprotonation affects the amide nitrogen group, geometry optimization leads to the most stable **Panto_b1** isomer that lies 27.6 (20.7) kJ mol^−1^ higher in energy relative to the global minimum **Panto_a1**, according to the more favorable energetics of proton removal from the carboxylic respect to amide group (Figure 2).

Deprotonation at such alternative site induces a significant impact on the hydrogen-bond network. In particular, **Panto_b1** is stabilized from the orientation of the terminal alcoholic unit donor toward the negatively charged nitrogen acceptor (r_N… HO4′_ = 1.844 Å), in addition to the bifurcated interaction of carbonyl oxygen with the hydroxyl units of secondary alcohol HO2′ (r_O1′… HO2′_ = 1.898 Å) and carboxylic group (r_O1′… HO1_ = 1.548 Å). 

A comprehensive presentation of amide deprotonated conformers, all found notably less stable than **Panto_a1**, is reported in Figure S6. Interestingly, relative to **Panto_b1**, a mismatched arrangement of the alcoholic hydroxyl groups destabilizes **Panto_b3** by only 0.7 (1.6) kJ mol^−1^, while the complete lack of hydrogen bonds involving the deprotonated amide in **Panto_b6** causes a drop in stability of 18.1 (15.2) kJ mol^−1^.

**Figure 3:**
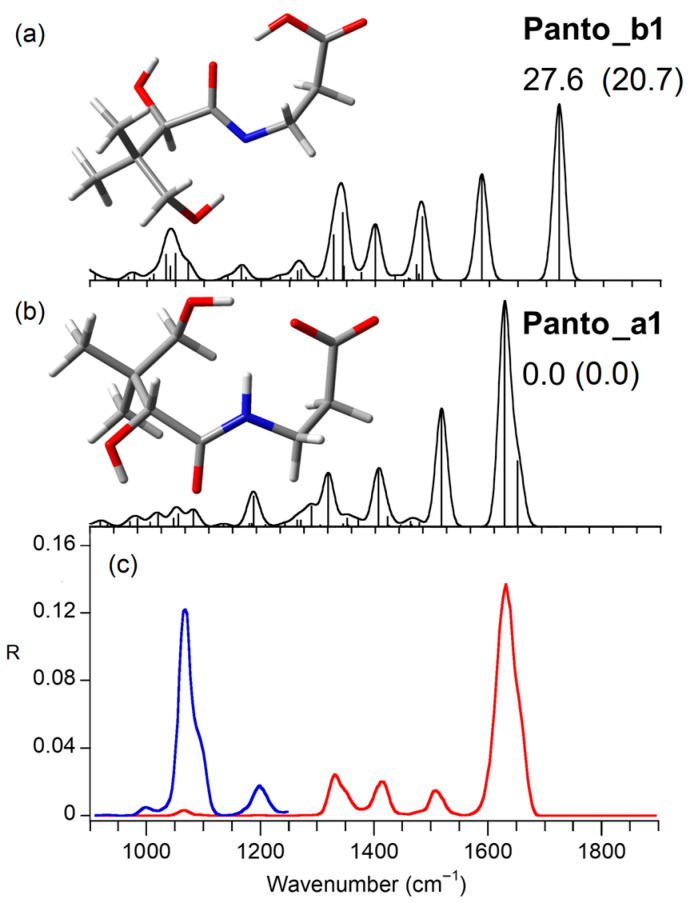
(**c**) Experimental IRMPD spectrum of deprotonated pantothenic acid [panto-H]^−^ (bottom, colored profiles); (**b**) computed IR spectrum of **Panto_a1** isomer; (**a**) computed IR spectrum of **Panto_b1** isomer. IR spectra and optimized structures are obtained at the B3LYP level. Relative free energies at 298 K in kJ mol^−1^ are computed at the B3LYP and MP2 level (in parentheses).

For structural and spectral assignments, the IRMPD spectrum of [panto-H]^−^ with the calculated IR absorption spectra of the low-lying isomers **Panto_a1** and **Panto_b1** are comparatively presented in Figure 3 in the 900–1900 cm^−1^ range. The position of the main IRMPD absorbances, together with the computed transitions of **Panto_a1** and a concise mode description, are listed in Table S2.

The linear IR spectra of structures deprotonated at either the carboxylic (**Panto_a1–6**) or the amide (**Panto_b1–6**) units are also presented in Figures S5 and S6, respectively. The calculated spectrum of **Panto_a1** explains fairly well the positions and the intensities of IRMPD bands of [panto-H]^−^, allowing us to assess the predominance of this geometry in the sampled gas-phase population. In particular, the dominant band at 1632 cm^−1^ encompasses (is well interpreted by) the amidic C1′ = O1′ (Amide I) and the asymmetric carboxylate stretches expected at 1650 and 1627 cm^−1^, respectively, which is in agreement with the somewhat broad and asymmetric form of the observed peak. The features at 1514, 1415, and 1334 cm^−1^ are well simulated by the NH bend (Amide II) at 1516 cm^−1^, the O2’H bend at 1405 cm^−1^, and the C1C2 stretch associated to the symmetric carboxylate stretch at 1318 cm^−1^ of **Panto_a1**, respectively. Indeed, the amide II vibrational mode expected around 1500–1550 cm^−1^ for CS geometry has been previously described as an excellent marker of the presence of protons on amide nitrogen atoms, providing an insight into the deprotonation site [32]. At still lower frequencies, also the weaker bands at 1198, 1074 with a shoulder at 1097 and 1001 cm^−1^ satisfactorily match with the bands predicted at 1187, 1081, 1055, and 1019 cm^−1^ that arise from the NH bend, C3N and C2′O2′ stretches, C3H2 and C2H2 rock modes, respectively. Incidentally, the absence of the COH bend at 1150 cm^−1^, a signature of a free terminal carboxylic group, further confirms deprotonation at the COOH unit. 

In agreement with its unfavorable energetic prediction, the calculated spectrum of **Panto_b1** does not agree with the experiment, disproving its presence in the assayed population. In fact, the most diagnostic bands due to stretches of the carboxylic C1 = O1 and the amide C1′ = O1′ carbonyl groups, which are predicted at 1722 and 1597 cm^−1^, respectively, are barely observed in the IRMPD spectrum (Figure 3).

### 2.4. Computed Structures and Spectral Assignments of [Ca(panto-H)]^+^

A similar analysis has been carried on [Ca(panto-H)]^+^ adducts of the conjugate base of pantothenic acid with divalent calcium ion. 

**Figure 4:**
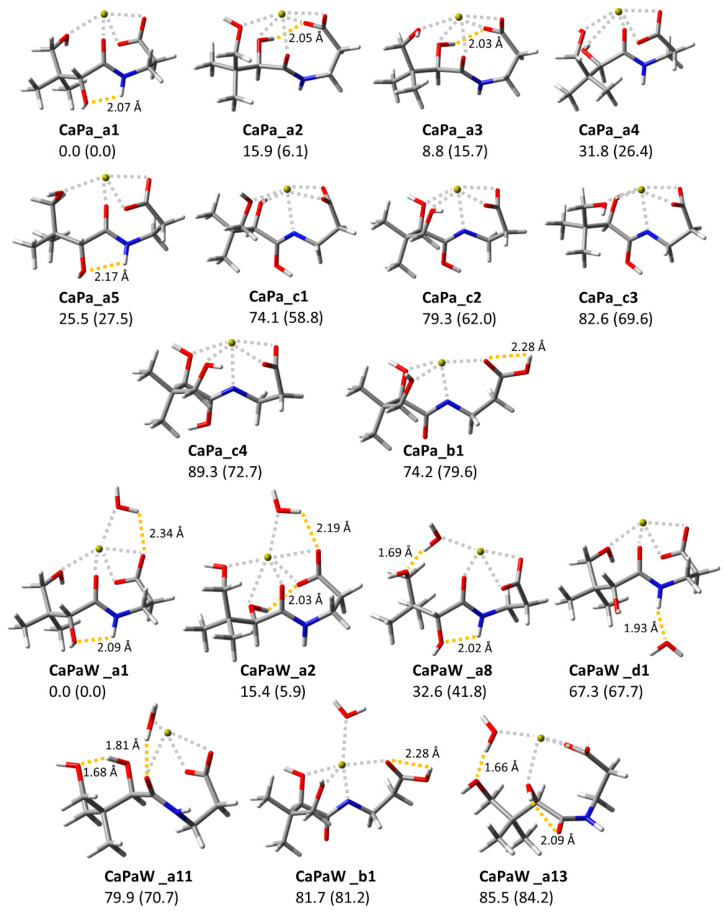
Structures of [Ca(panto-H)]^+^ and [Ca(panto-H)(H_2_O)]^+^ optimized at the B3LYP/6-311++G(d,p) level. Hydrogen bonds are reported with yellow dashed lines together with their length, while gray dashed lines indicate metal bonds. Relative free energies at 298 K in kJ mol^−1^ are computed at the B3LYP and MP2 level (in parentheses).

In previous work, a systematic characterization of the chelating strength and binding preferences in complexes of alkali and alkaline earth metal ions with small peptides have been carried out relying on spectroscopic, thermochemical, and theoretical approaches, showing a preference for oxygen binding [50,52]. In particular, spectroscopic evidence has confirmed the weak–intermediate coordination properties of Ca(II) ions that prefer to adopt CS chelation by the amide carbonyl oxygens, along with some admixture of an iminol binding motif, which is formed by the deprotonation of amide nitrogen [32,50,58,61]. In this context, the structural modification of pantothenate determined by calcium binding has been explored in the sampled [Ca(panto-H)]^+^ ion. The relevant thermodynamic data are presented in Table S3, while the whole set of structures is reported in Figure 4. Potential candidates, where the metal ion interacts with the O-atoms of a carboxylate unit and either the amide carbonyl (**CaPa_a1–5**) or the iminol nitrogen atom (**CaPa_c1–4)**, have been identified, each in turn originating a family of low-lying conformers by rotation around single bonds of the flexible pantothenate skeleton.

In addition, calcium coordination at deprotonated amide nitrogen has allowed us to describe a considerably less favorable isomer (**CaPa_b1**), bearing an intact carboxylic unit in a cis configuration.

Overall, the same stability order is found by employing both B3LYP(-D3) and MP2, with the ground state conformer **CaPa_a1** stabilized by a tetradentate interaction with the oxygens of carboxylate (O1a, O1b), carbonyl (O1′), and primary alcoholic (O4′) groups, along with an HN…O2′H (r_HN…HO2′_ = 2.075 Å) hydrogen bond. Only in the case of the pentadentate **CaPa_a3** and **CaPa_a2** does a modest change in the energy values cause a switch in the energy ordering, with the latter conformer located at 6.1 kJ mol^−1^ above **CaPa_a1** at single point MP2 calculations (Figure 4 and Table S3).

In all geometries, the binding of Ca(II) ions to pantothenate ligand is anchored by additional chelation to one or both alcoholic hydroxyl groups, thus yielding tetra- and pentadentate coordination. In particular, while the lowest lying structure **CaPa_a1** can be visualized as a distorted square-based pyramid, in **CaPa_a2**, an additional coordination site can be identified rising from the secondary alcoholic oxygen (O2′), which is therefore no more involved in hydrogen bonding.

Noteworthily, the 10 kJ mol^−1^ higher stability of this rotamer predicted at the MP2 level suggests that dispersion corrections play a (small) positive role by appropriately simulating the interaction of calcium with alcoholic oxygens [28,34,36].

In the case of **CaPa_a3**, at 8.8 (15.7) kJ mol^−1^ above the ground state, the rotation about the C3′C4′ bond weakens the contact between the O2′ hydroxyl oxygen and calcium ion. Other less stable arrangements, at 25.5 (27.5) and 31.8 (26.4) kJ mol^−1^ relative to **CaPa_a1**, are respectively found in **CaPa_a5** and **CaPa_a4**, the latter form lacking the O2′ interaction with the metal center.

**Figure 5:**
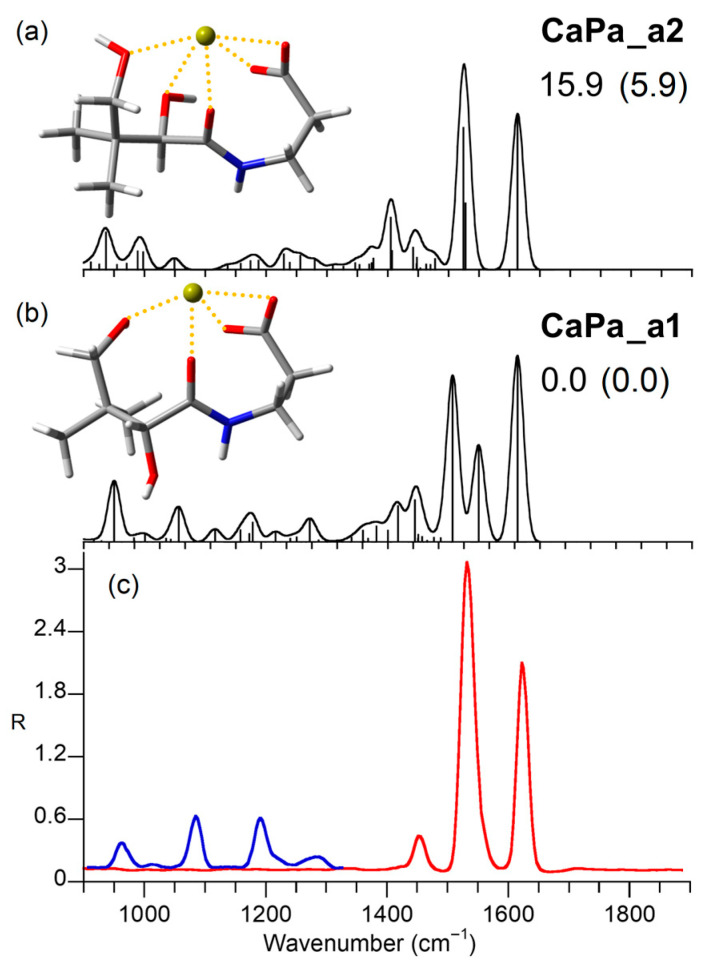
(**c**) IRMPD spectrum of [Ca(panto-H)]^+^ in the fingerprint range obtained submitting the ions either to 1 s of FEL light (blue profile) or to 500 ms using one attenuator (red profile). The calculated spectra of the lowest lying structures (**b**) **CaPa_a1** and (**a**) **CaPa_a2** are reported in black together with their optimized geometry and relative free energies (298 K) at the B3LYP and MP2 (in bracket) levels in kJ mol^−1^.

A comparison of the IRMPD spectrum of [Ca(panto-H)]^+^ with the computed IR spectra of the lowest energy structures **CaPa_a1** and **CaPa_a2** is reported in Figure 5, while the calculated spectra of highest energy conformers are shown in Figures S7 and S8. Indeed, elements of both **CaPa_a1** and **CaPa_a2** can satisfactorily explain the experimental spectrum, indicating a mixture of conformers in the gas-phase population of [Ca(panto-H)]^+^ (Figure 5). Table 1 illustrates the position of the main IRMPD absorptions, the computed transitions of the **CaPa_a1** and **CaPa_a2** rotamers, and a short mode description.

The intense band observed at 1625 cm^−1^ is in good agreement with the C1′O1′ stretching (Amide I) calculated at 1613 and 1614 cm^−1^ for **CaPa_a1** and **CaPa_a2**, respectively.

The prominent IRMPD signal at 1530 cm^−1^ is somewhat trickier to assign. In particular, **CaPa_a2** simulates the experiment rather well, with the NH bend (Amide II) at 1528 cm^−1^ and the asymmetric carboxylate stretch at 1524 cm^−1^, while the same vibrational modes in **CaPa_a1** are spaced by 42 cm^−1^, the NH bend being calculated at 1549 cm^−1^ and the asymmetric carboxylate stretch at 1507 cm^−1^. In the tetracoordinated **CaPa_a1** form, a smaller degree of coordination/binding that may strengthen calcium–pantothenate interaction, together with an NH intramolecular hydrogen bond can likely be the origin of the slight red shift of asymmetric carboxylate stretch along with the blue shift of NH bending vibration with respect to **CaPa_a2** [23,62].

However, the possible contribution of **CaPa_a1** to the assayed gas-phase population can be inferred when considering that the experimental band at 1530 cm^−1^ is somewhat broad (FWHM = 30 cm^−1^) with a shoulder on the blue side. This result may be arguably due to the presence of an admixture of contributing tetra- and pentacoordinated structures, with a major contribution of **CaPa_a2**, which is more compatible with the MP2 than both B3LYP and B3LYP-D3 prediction.

Interestingly, the observed frequencies of Amide I and Amide II modes, respectively red and blue shifted relative to an uncomplexed amide group, agree with previous evidence on Ca-coordinated peptide adducts [62].

In addition, the IRMPD band at 1450 cm^−1^ encompasses the CH_2_ scissoring, CH_3_ umbrella, and C1C2 and symmetric carboxylate stretches, predicted at 1417 cm^−1^ (**CaPa_a1**) and 1405 cm^−1^ (**CaPa_a2**), together with O2′H bend (**CaPa_a1**), and C3H_2_ scissoring (**CaPa_a1**), calculated at 1445 and 1441 cm^−1^, respectively. Finally, the weaker peaks at 1285, 1192, 1085, and 965 cm^−1^ are due to contributions by **CaPa_a1**, with NH bend and CH bends at 1271 cm^−1^; O2′H bend and C2H_2_ twist at 1178 cm^−1^; C2′O2′ stretch and CH_2_ rock at 1056 cm^−1^; C4′O4′ coupled with a C2′O2′ stretch at 950 cm^−1^, and by **CaPa_a2**, with C4′O4′ coupled with a C2′O2′ stretch and CH_3_ bending at 937 and 989 cm^−1^. In summary, no other arrangement fulfills as well the matching with the IRMPD spectrum, thus confirming Ca(II) to prefer CS binding to carboxylate oxygens and anchoring carbonyl and alcoholic hydroxyl units of deprotonated pantothenic acid. A fully oxygenated coordination pattern was also disclosed by X-ray crystallographic analysis of calcium bromide salt of pantothenic acid [63].

Conversely, the iminol structural motif present in the less stable isomer **CaPa_c1** and all other representative conformers of the series, **CaPa_c2–4**, has direct bearing on their IR spectra that are in marked contrast with the experiment (Figure S8). In particular, the intense bands at 1700 and 1150 cm^−1^, which comprise the CN stretch coupled with C1′O1′stretch, and the O1′H bending mode, respectively, are not consistent with experimental evidence. Any significant contribution of the less stable isomer **CaPa_b1** can be also discarded, based on the following: (i) the absence of any resonance in the range 1500–1600 cm^−1^, which is in contrast with the pronounced feature found in the IRMPD spectrum; and (ii) the calculated bands at 1647, 1635, and 1316 cm^−1^, attributed to amide C1′ = O1′ stretch, carboxylic C1=O1 stretch coupled with O1H bend, and CN stretch coupled with C2′H bend, respectively, all by no means observed in the IRMPD profile.

### 2.5. Computed Structures and Spectral Assignments of [Ca(panto-H)(H_2_O)]^+^

The propensity of alkaline earth metals to hold some water molecules is well recognized, as well as their extensive dehydration when interacting with the carbonyl oxygens of transport, membrane, and modulator proteins. Typically, the relatively large and hard Ca(II) ion realizes geometries with coordination number of 7, where side chain carboxylate, backbone carboxamide, alcohol functionalities, and water molecules are documented to fulfill the preferred coordination sphere [64].

Here, we have examined the effect of micro hydration with a single water molecule on the structural and spectroscopic features of the calcium complex encased in vitamin B5. The aim is to ascertain the preferred binding site of water and any possible solvent promoted isomerization. Previous evidence on the monohydrated complexes of Pb(II) ion with deprotonated proline has revealed that solvation occurring in the hexapole storage allows a proton transfer isomerization with a change in lead coordination from deprotonated amino nitrogen and carbonyl oxygen to the two carboxylate oxygen atoms [65]. A solvation-induced switch from iminol to a charge-solvated binding pattern has been also observed upon mono-hydration of the Ni(II)-triglycine complex [66]. The effect of micro-hydration on neutral vit B5 has been also evidenced to catalyze tautomerism from the more stable keto-amine to the enol-enamine form by lowering the activation energy by about 90 kJ mol^−1^ [67].

**Figure 6:**
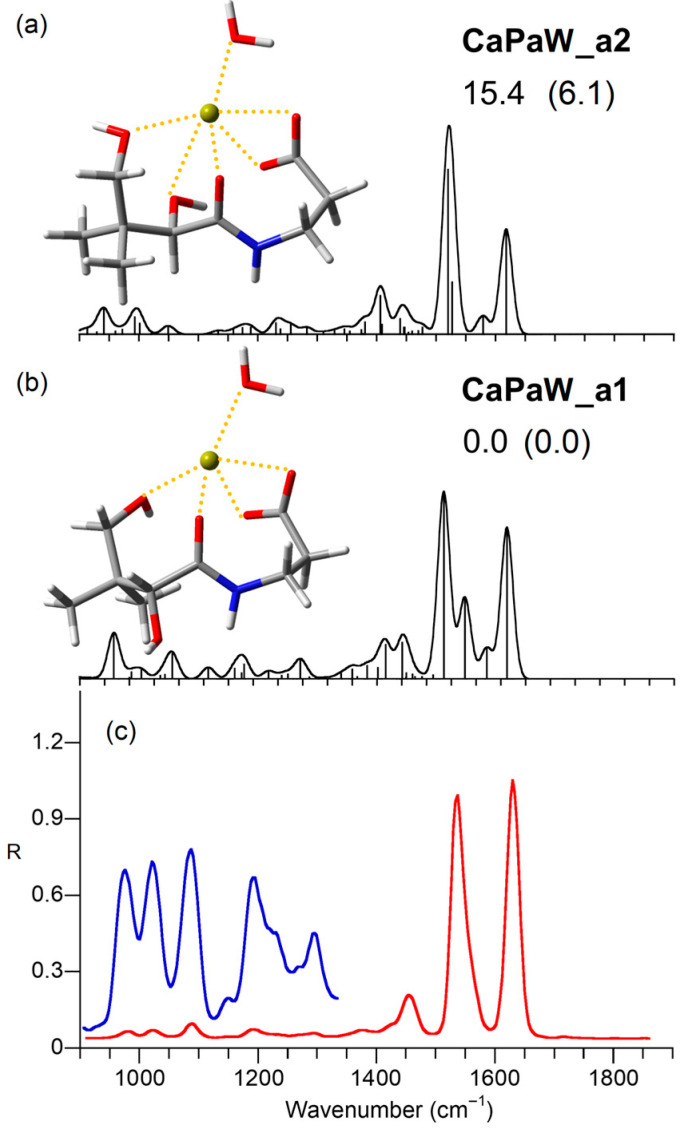
(**c**) Experimental IRMPD spectrum of [Ca(panto-H)(H_2_O)]^+^ (bottom, colored profiles) and computed IR spectra of isomers (**b**) **CaPaW_a1** and (**a**) **CaPaW_a2** obtained at the B3LYP level. Optimized structures at the B3LYP level are reported together with free energies at 298 K in kJ mol^−1^ computed at the B3LYP and MP2 level (in parentheses).

The IRMPD spectrum of the monohydrated complex, [Ca(panto-H)(H_2_O)]^+^, which closely resembles that of bare [Ca(panto-H)]^+^, is compared with the calculated IR spectra of the two most stable tetradentate (**CaPaW_a1**), the global minimum, and pentadentate (**CaPaW_a2**) structures in Figure 6 and a selection of optimized geometries is reported in Figure 4. Thermodynamic data regarding all the sampled structures are presented in Table S4, while their calculated spectra are shown in Figures S9, S10, and S11. Both geometries, which differ by only 5.9 kJ mol^−1^, are singly solvated versions of the lowest energy bare adduct, [Ca(panto-H)]^+^, which is stabilized by extra coordination of calcium ion with an intact water molecule that in turn forms hydrogen bonds to a carboxylate oxygen of pantothenate (r_O1_^−^_…HO_ ~ 2.2 Å). This type of solvation is more favorable relative to different skeletal arrangements such as in the following: (i) isomers **CaPaW_a8/11** and **13**, where a bridging water molecule is simultaneously coordinating Ca(II) ion and forming a hydrogen bond with the primary alcohol O4′H and carbonyl O1′ acceptors; (ii) **CaPaW_d1**, where the amide NH donor interacts with an external water molecule; (iii) **CaPaW_b1,** with deprotonated amide nitrogen binding to Ca(II) ion. These structures are found to lie 41.8, 84.2, 67.7, and 81.2 kJ mol^−1^ higher in energy relative to the lowest energy structure **CaPaW_a1**, respectively (Figure 4). Moreover, arrangements where a water molecule is placed external to the metal coordination sphere, occurring for example in **CaPaW_d1**, are less likely and have been hardly observed under our experimental conditions [68]. As a result, any significant contribution of these isomers to a thermally averaged gas-phase population can be ruled out.

The experimental spectrum of [Ca(panto-H)(H_2_O)]^+^ is consistent with calculations, clearly showing that the penta- and hexa-coordinate canonical, charge-solvated geometries of **CaPaW_a1** and **CaPaW_a2** are the main contributors, with water and alcoholic OH groups providing the extra stability to calcium ion chelation by the amide carbonyl and carboxylate oxygens.

In particular, the dominant absorption at 1630 cm^−1^ is assigned to C1′O1′ stretch, and the intense band at 1536 cm^−1^ encompasses NH bend and asymmetric carboxylate stretching modes, respectively, which are predicted at the same frequencies in both **CaPaW_a1** and **CaPaW_a2** (see Table S5 of the Supplementary Materials) in a similar way as already detailed for [Ca(panto-H)]^+^. Unfortunately, in this IR range, the only vibrational mode characteristic of the water molecule is the H_2_O scissoring, which is located at around 1580 cm^−1^ for both rotamers, an area poorly indicative due to the presence of the two intense signals previously described. Moreover, the presence of adventitious water vapor may cause rather weaker laser intensity, lowering the IRMPD activity at this frequency [68].

The band at 1449 cm^−1^ comprises resonances associated with O2′H bending, CH_2_ scissoring modes, and the symmetric carboxylate stretching. The region below 1400 cm^−1^ appears considerably enriched when compared to the IRMPD spectrum of [Ca(panto-H)]^+^ as a consequence of a low energy threshold for water departure from the [Ca(panto-H)(H_2_O)]^+^ complex [31]. Thus, the efficient photofragmentation allows us to highlight the complexity of the absorption pattern in this region, which is in good agreement with the numerous predicted features. Noteworthily, the mismatch with the IRMPD spectrum of the spectroscopic signatures of a bridging water interaction, such as those envisaged in **CaPaW_a11/a13** isomers, i.e., the water scissoring coupled with either the C-O1 or C-O4′ stretching modes predicted beyond 1650 cm^−1^, is notably absent in the experimental spectrum, leading us to exclude these energetically disfavored structures (see Figure S11).

## 3. Materials and Methods

### 3.1. Sample Preparation

All solvents and reagents including calcium D-pantothenate (3-[(2R)-2,4-dihydroxy-3,3-dimethylbutanamido] propanoic acid), vitamin B5, were research grade commercial products (Sigma-Aldrich s.r.l., Milan, Italy) used as supplied. The calcium complexes, [Ca(panto-H)]^+^ (*m*/*z* 258) and the singly hydrated ion [Ca(panto-H)(H_2_O)]^+^ (*m*/*z* 276), were generated by ESI in positive mode by direct infusion of 2 µM of vitamin B5 dissolved in water/methanol (2:1 *v*/*v*) at a flow rate of 2 µL min^−1^. Deprotonated pantothenic acid, [panto-H]^−^ (*m*/*z* 218), was generated by submitting to ESI in negative mode the above solution, with 2% ammonia to assist deprotonation. Accurate ion mass measurements were carried out in the cell analyzer of using a Bruker BioApex Fourier Transform ion cyclotron resonance (FT-ICR) [69] mass spectrometer (Bruker Daltonics GmbH, Bremen, Germany) equipped with an Apollo I electrospray ionization (ESI) source and a 4.7 T superconducting magnet (FT-ICR lab, Sapienza Università di Roma) with external calibration (Δm < 2 ppm).

### 3.2. MS and IRMPD Spectroscopy

The IRMPD spectra of mass-selected ions were recorded in the mid-IR/fingerprint range (900–1900 cm^−1^) using the tunable (5–25 µm) free electron laser (FEL) source at the Centre Laser Infrarouge d′Orsay (CLIO) facility [70]. The FEL electron energy was set at 40.0 and 44.4 MeV in two separate runs to optimize the laser power at approximately 900–1100 mW in the frequency range of interest. The FEL source delivers 9 µs long trains of macropulses at a repetition rate of 25 Hz, each containing 600 micropulses (0.5–3 ps long). Typical macropulse energies were 40 mJ. The IR-FEL spectral width (full-width at half-maximum, fwhm) was less than 0.5% of the central wavelength.

IRMPD experiments were performed by coupling a CLIO beam to a modified mass spectrometer, either a Paul ion trap Bruker Esquire 3000+ [71] in the case of [panto-H]^−^ (*m*/*z* 218) and a hybrid Fourier transform ion-cyclotron resonance (FT-ICR) tandem mass spectrometer (Apex-Qe Bruker Daltonics) [72], equipped with a 7.0 T actively shielded magnet and a quadrupole–hexapole interface for mass-filtering and collisional cooling for 300 ms with Ar as buffer gas, for [Ca(panto-H)]^+^ (*m*/*z* 258) and [Ca(panto-H)(H_2_O)]^+^ (*m*/*z* 276). The singly hydrated complex was formed in the storage hexapole.

In the ion trap, [panto-H]^−^ ions were accumulated for 5–10 ms and mass-selected prior to IR irradiation, whereas in the latter setup, the calcium complexes were mass selected in the quadrupole mass filter stage and then accumulated in an rf hexapole trap for 50 ms. All the assayed ions were irradiated for 0.3–1 s, except for the hydrated [Ca(panto-H)(H_2_O)]^+^ complex for which softer laser power parameters were applied, i.e., a shorter IR-FEL irradiation time of 250–500 ms. In order to minimize saturation of the most pronounced bands, IRMPD spectra were also recorded using 1–3 attenuators, each decreasing the power by a factor of three. Sequential absorption of multiple resonant photons, associated with intramolecular vibrational energy redistribution, may eventually lead to a wavelength-dependent photofragmentation process [15]. IRMPD spectra were collected by recording the photodissociation yield R, which is defined as −ln[*I_P_*/(*I_P_* + Σ*I_F_*)], where *I_P_* and *I_F_* are the abundances of the parent ion and of the fragment ions, respectively, as a function of the IR photon energy [73]. At each selected wavenumber, four mass spectra were registered and averaged.

### 3.3. Computational Details/Theoretical Calculations

Conformational analysis was performed using the Conformer Distribution tool as implemented in the Spartan’14 software (Wavefunction Inc., Irvine, CA, USA) with the pseudoempirical method PM6 starting from tautomeric structures with a proton on either the nitrogen or oxygen of the amidic group. The whole set of obtained conformers, ca. 80 for both [panto-H]^−^ and [Ca(panto-H)]^+^ ions, were subsequently optimized at the B3LYP/6-31 + G * level of theory using Gaussian 09 rev.D01 (Gaussian Inc., Wallingford, CT, USA) [74].

The structures inside an energetic range of 20 kJ mol^−1^ have been finally reoptimized at the B3LYP/6-311++G(d,p) level. The structures presented for [Ca(panto-H)(H_2_O)]^+^ have been prepared by adding a water molecule to the coordination sphere of calcium in the [Ca(panto-H)]^+^ lowest lying optimized geometries. The whole set of structures was newly optimized at the B3LYP-D3/6-311++G(d,p) level to evaluate the effect of dispersion. Refined electronic energies were obtained from single-point MP2(full)/6-311++G(d,p) calculations using the B3LYP geometries. The relative enthalpies and Gibbs free energies (298 K) at the MP2 level were obtained by a combination of the single-point MP2 electronic energies and B3LYP/6-311++G(d,p) thermal corrections (298 K).

Frequency calculations were performed at the B3LYP(-D3)/6-311++G(d,p) levels. The spectra calculated by using the dispersion corrected functional B3LYP-D3 are not herein reported being superimposable to the B3LYP ones. Calculated IR spectra in the fingerprint range (900–1900 cm^−1^) were uniformly scaled using a best fit factor (0.974) [42,57]. The relative energies of the low-lying structures at 0K, enthalpies, and Gibbs free energies at 298 K were achieved by applying zero-point and thermal energy correction. A Lorentzian profile with a full-width at half-maximum (fwhm) of 12 cm^−1^ was adopted in order to better simulate the experimental peak broadening.

## 4. Conclusions

A joint investigation based on vibrational spectra in the fingerprint range and theoretical calculations at both B3LYP and MP2 levels of theory using the 6-311++G(d,p) basis set allows us to thoroughly characterize the conjugate base of pantothenic acid as well as its bare and monohydrated calcium complexes obtained by electrospray ionization as free species in the gas phase. Pantothenate ion was shown to be deprotonated at the carboxylic group, and the calcium complexes adopt a charge-solvated multidentate structure, which confirms the preferred Ca(II) binding mode already described for small peptides. In the deprotonated species [panto-H]^−^, only the lowest energy structure is observed, which is stabilized by a folded arrangement favoring hydrogen bonds between the carboxylate group and both the amide and primary alcohol hydrogens, while a carbonyl oxygen is engaged with the secondary hydroxyl unit. In both the [Ca(panto-H)]^+^ complex and its singly hydrated adduct [Ca(panto-H)(H_2_O)]^+^, it arises that their IRMPD spectra are very similar and are both assigned as arising from a mixture of two close-lying geometries. In the former complex, the ground state structure is stabilized by Ca(II) ion chelation involving oxygen atoms belonging to carboxylate, carbonyl, and primary alcohol functionalities in a tetradentate arrangement, while the amide NH forms a hydrogen bond with the secondary alcohol. [Ca(panto-H)]^+^ and [Ca(panto-H)(H_2_O)]^+^ have at most five- and six-coordination spheres, respectively.

The presence of water has little effect on the IRMPD spectra and calculated low energy structures present an additional metal–oxygen bond involving the water ligand. This evidence excludes here any isomerization reaction that could be catalyzed by the additional presence of a water molecule, as previously described for some monohydrated (transition) metal complexes. 

The combined experimental and computational approach applied in this study on vitamin B5 is part of ongoing research aiming to the structural elucidation of individual metabolites and provides further molecular insight into metal-ion binding motifs to physiologically important molecules.

## Figures and Tables

**Table 1:** Observed IRMPD bands and calculated vibrational frequencies for the lowest lying structures **CaPa_a1** and **CaPa_a2** of [Ca(panto-H)]^+^.

Calculated ^a,b^			
IRMPD ^a^	CaPa_a1	CaPa_a2	Vibrational Mode
965	950 (126)	937 (77)	C4′O4′ stretch + C2′O2′ stretch + CH_3_ bend
		989 (146)	C4′O4′ stretch + CH_3_ bend
		998 (35)	C1′C2′ stretch + C3N stretch
1010			
1085	1056 (70)		C2′O2′ stretch + CH_2_ (all) rock
1192	1178 (40)		O2′H bend + C2H_2_ twist
1285	1271 (47)		NH bend + C2′H bend + C3H bend
1379	1381 (32)		O4′H bend + C4′H bend + CH_3_ umbrella
1450	1417 (74)	1405 (107)	C2H_2_ scissor + C1C2 stretch + sym carboxylate stretch + CH_3_ umbrella
		1407 (38)	C2H_2_ scissor + C3H_2_ scissor + CH_3_ umbrella
	1445 (86)		O2′H bend + C4′H2 wagging
		1441 (45)	C2H_2_ scissor + C3H_2_ scissor
1533	1507 (342)	1524 (291)	antisym carboxylate stretch + C2H_2_ twist
	1549 (200)	1528 (136)	NH bend (Amide II) + CN stretch
1625	1613 (384)	1614 (321)	C1′O1′ stretch (Amide I)

^a^ in cm^−1^. ^b^ intensities in parentheses in km mol^−1^. Vibrations with intensities lower than 30 km mol^−1^ are not reported.

## Data Availability

The data presented in this study are available on request from the corresponding author.

## Supplementary Materials

Supplementary materials can be found at https://www.mdpi.com/1422-0067/22/2/692/s1.
