# Supplementary material for: Molecular Properties of Bare and Microhydrated Vitamin B5–Calcium Complexes
Source: Int J Mol Sci. 2021 Jan 12;22(2):692. doi: 10.3390/ijms22020692 (PMC7826572; doi:10.3390/ijms22020692)
Supplement: Supplementary file 1 [file ijms-22-00692-s001.zip › ijms-supplementary_05.01.2021.docx]

Supporting Information

Molecular properties of bare and microhydrated vitamin B5 - calcium complexes

Davide Corinti^1^, Barbara Chiavarino^1^, Debora Scuderi2, Caterina Fraschetti^1^, Antonello Filippi^1^, Simonetta Fornarini^1^, Maria Elisa Crestoni^1,^*

^1^ Dipartimento di Chimica e Tecnologie del Farmaco, Università di Roma “La Sapienza,” Piazzale Aldo Moro, 5, I-00185 Roma, Italy; [davide.corinti@uniroma1.it](mailto:davide.corinti@uniroma1.it) (D.C.); [barbara.chiavarino@uniroma1.it](mailto:barbara.chiavarino@uniroma1.it) (B.C.); [caterina.fraschetti@uniroma1.it](mailto:caterina.fraschetti@uniroma1.it) (C.F.); [antonello.filippi@uniroma1.it](mailto:antonello.filippi@uniroma1.it) (A.F.); [simonetta.fornarini@uniroma1.it](mailto:simonetta.fornarini@uniroma1.it) (S.F.); [mariaelisa.crestoni@uniroma1.it](mailto:mariaelisa.crestoni@uniroma1.it) (M.E.C.)

^2^ Institut de Chimie Physique (UMR8000), CNRS, Université Paris-Saclay, 91405, Orsay, France; [debora.scuderi@universite-paris-saclay.fr](mailto:debora.scuderi@universite-paris-saclay.fr) (D.S.)

***** Correspondence: mariaelisa.crestoni@uniroma1.it; Tel +39 06 4991 3596 (M.E.C.)

**Table of contents**

**p. S3** **Figure S1** ESI mass spectra of a calcium D-panthotenate solution in water/methanol (2:1 v/v) recorded in either (a) positive or (b) negative ion mode. One can highlight the presence in the positive spectrum of [panto+H]^+^ and [panto+Na]^+^ at *m/z* 219.8 and 241.9, respectively and of the calcium complexes [Ca(panto-H)]^+^ and [Ca(panto-H)(H_2_O)]^+^ at *m/z* 257.8 and 275.8, respectively. The signal in the negative spectrum at *m/z* 217.7 is attributed to [panto-H]^-^.

**p. S4 Figure S2.** Positive ESI mass spectra recorded upon selection of the [Ca(panto-H)(H_2_O)]^+^ ion (*m/z* 276), in a FT-ICR mass spectrometer (Bruker, Apex-Qe), (b) before and (a) after irradiation with CLIO FEL light on resonance at 1460 cm^-1^.

**p. S5 Figure S3.** Positive ESI mass spectra recorded upon selection of the [Ca(panto-H)]^+^ ion (*m/z* 258), in a FT-ICR mass spectrometer (Bruker, Apex-Qe), (b) before and (a) after irradiation with CLIO FEL light on resonance at 1630 cm^-1^.

**p. S6 Figure S4.** Negative ESI mass spectra recorded upon selection of deprotonated pantothenic acid [panto-H]^-^ ion (*m/z* 218), in a Bruker Esquire Paul ion trap mass spectrometer (Bruker, Esquire 3000+), (b) before and (a) after irradiation with CLIO FEL light on resonance at 1510 cm^-1^.

**p. S7 Figure S5**. (a) IRMPD spectrum of [panto-H]^-^ together with (b-g) calculated spectra at the B3LYP/6-311++G(d,p) level. Spectra were scaled by a factor of 0.974. Relative free energies at 298 K are reported in kJ mol^-1^ at the B3LYP and MP2 (in parentheses) level.

**p. S8 Figure S6**. (h) IRMPD spectrum of [panto-H]^-^ together with (i-o) calculated spectra at the B3LYP/6-311++G(d,p) level. Spectra were scaled by a factor of 0.974. Relative free energies at 298 K are reported in kJ mol^-1^ at the B3LYP and MP2 (in parentheses) level

**p. S9 Figure S7**. (a) IRMPD spectrum of [Ca(panto-H)]^+^ together with (b-f) calculated spectra at the B3LYP/6-311++G(d,p) level. Spectra were scaled by a factor of 0.974. Relative free energies at 298 K are reported in kJ mol^-1^ at the B3LYP and MP2 (in parentheses) level.

**p. S10 Figure S8**. (g) IRMPD spectrum of [Ca(panto-H)]^+^ together with (h-i) calculated spectra at the B3LYP/6-311++G(d,p) level. Spectra were scaled by a factor of 0.974. Relative free energies at 298 K are reported in kJ mol^-1^ at the B3LYP and MP2 (in parentheses) level.

**p. S11 Figure S9**. (a) IRMPD spectrum of [Ca(panto-H)(H_2_O)]^+^ together with (b-g) calculated spectra at the B3LYP/6-311++G(d,p) level. Spectra were scaled by a factor of 0.974. Relative free energies at 298 K are reported in kJ mol^-1^ at the B3LYP and MP2 (in parentheses) level.

**p. S12 Figure S10**. (h) IRMPD spectrum of [Ca(panto-H)(H_2_O)]^+^ together with (i-m) calculated spectra at the B3LYP/6-311++G(d,p) level. Spectra were scaled by a factor of 0.974. Relative free energies at 298 K are reported in kJ mol^-1^ at the B3LYP and MP2 (in parentheses) level

**p. S13 Figure S11**. (n) IRMPD spectrum of [Ca(panto-H)(H_2_O)]^+^ together with (o-s) calculated spectra at the B3LYP/6-311++G(d,p) level. Spectra were scaled by a factor of 0.974. Relative free energies at 298 K are reported in kJ mol^-1^ at the B3LYP and MP2 (in parentheses) level

**p. S14 Table S1.** Thermodynamic data (kJ mol^-1^) for the most stable structures of [panto-H]^-^ calculated at either the B3LYP(-D3) or MP2 levels.

**p. S15 Table S2.** Observed IRMPD bands and calculated vibrational frequencies for the lowest lying structure **Panto_a1** and **Panto_b1** of deprotonated pantothenic acid, [panto-H]^-^.

**p. S16 Table S3.** Thermodynamic data (kJ mol^-1^) for the most stable structures of [Ca(panto-H)]^+^ calculated at either the B3LYP(-D3) or MP2 levels.

**p. S17 Table S4.** Thermodynamic data (kJ mol^-1^) for the most stable structures of [Ca(panto-H)(H_2_O)]^+^ calculated at either the B3LYP(-D3) or MP2 levels.

**p. S18 Table S5.** Observed IRMPD bands and calculated vibrational frequencies for the lowest lying structures **CaPaW_a1** and **CaPaW_a2** of [Ca(panto-H)(H_2_O)]^+^.


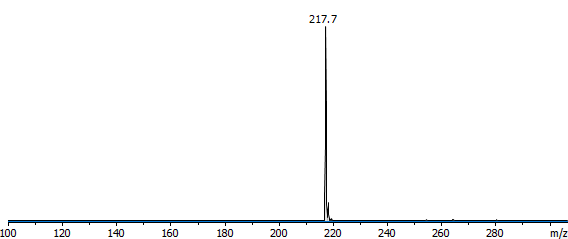

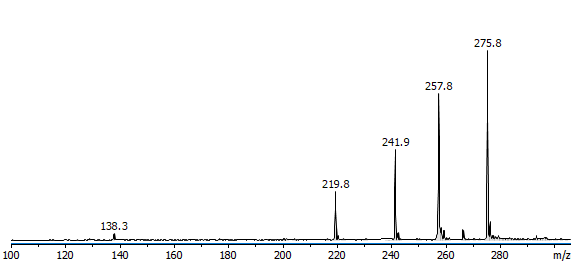


(a)

(b)

**Figure S1** ESI mass spectra of a calcium D-panthotenate solution in water/methanol (2:1 v/v) recorded in either (a) positive or (b) negative ion mode. One can highlight the presence in the positive spectrum of [panto+H]^+^ and [panto+Na]^+^ at *m/z* 219.8 and 241.9, respectively and of the calcium complexes [Ca(panto-H)]^+^ and [Ca(panto-H)(H_2_O)]^+^ at *m/z* 257.8 and 275.8, respectively. The signal in the negative spectrum at *m/z* 217.7 is attributed to [panto-H]^-^.


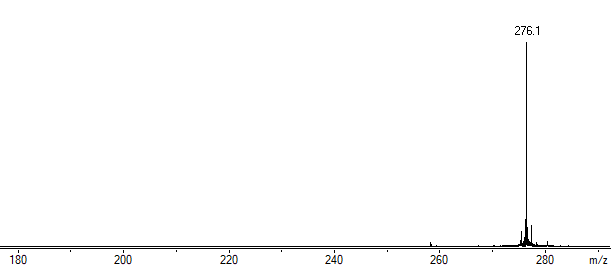

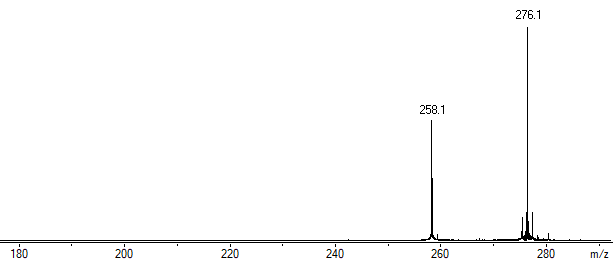


-H_2_O

(b)

(a)

**Figure S2.** Positive ESI mass spectra recorded upon selection of the [Ca(panto-H)(H_2_O)]^+^ ion (*m/z* 276), in a FT-ICR mass spectrometer (Bruker, Apex-Qe), (b) before and (a) after irradiation with CLIO FEL light on resonance at 1460 cm^-1^.


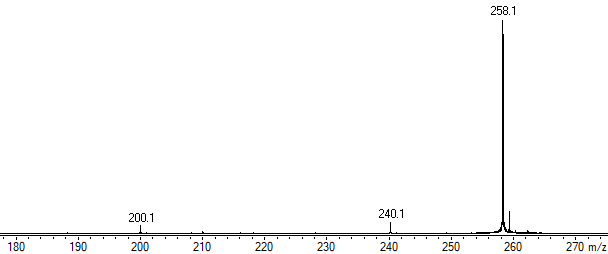

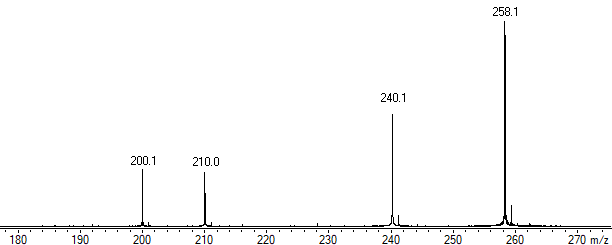


-H_2_O

-H_2_CO

-H_2_C_2_O_2_

(b)

(a)

**Figure S3.** Positive ESI mass spectra recorded upon selection of the [Ca(panto-H)]^+^ ion (*m/z* 258), in a FT-ICR mass spectrometer (Bruker, Apex-Qe), (b) before and (a) after irradiation with CLIO FEL light on resonance at 1630 cm^-1^.

(b)


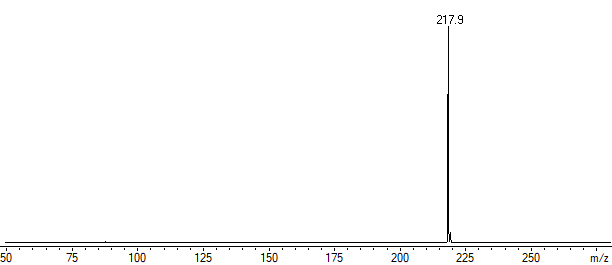

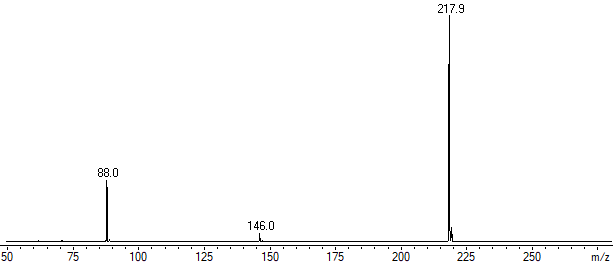


[(β-Alanine)^-^H]^-^

-(CO_2_ + CO)

(a)

**Figure S4.** Negative ESI mass spectra recorded upon selection of deprotonated pantothenic acid [panto-H]^-^ ion (*m/z* 218), in a Bruker Esquire Paul ion trap mass spectrometer (Bruker, Esquire 3000+), (b) before and (a) after irradiation with CLIO FEL light on resonance at 1510 cm^-1^.

**Panto_a4**

17.9 (16.4)


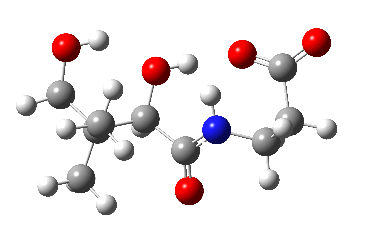


**Panto_a5**

10.6 (17.5)


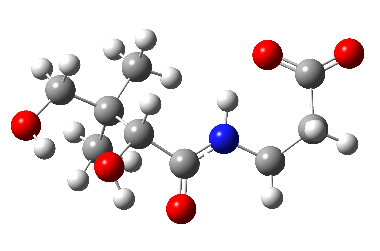


**Panto_a3**

8.2 (5.6)


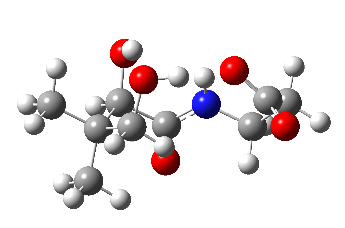


**Panto_a2**

0.9 (1.5)


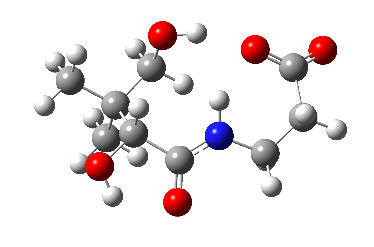


**Panto_a1**

0.0 (0.0)

**Panto_a6**

19.3 (18.2)


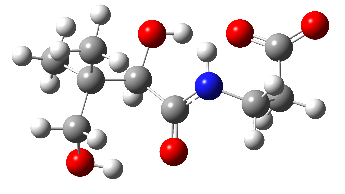

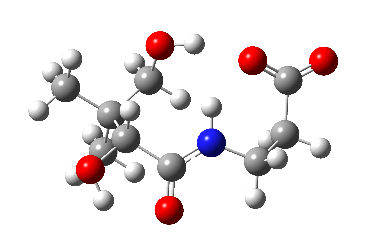


(a)

(b)

(c)

(d)

(f)

(e)

(g)

**Figure S5**. (a) IRMPD spectrum of [panto-H]^-^ together with (b-g) calculated spectra at the B3LYP/6-311++G(d,p) level. Spectra were scaled by a factor of 0.974. Relative free energies at 298 K are reported in kJ mol^-1^ at the B3LYP and MP2 (in parentheses) level.

**Panto_b1**

27.6 (20.7)

**Panto_b6**

45.7 (35.8)

**Panto_b5**

37.3 (33.9)

**Panto_b4**

29.7 (25.4)

**Panto_b2**

28.5 (21.1)

**Panto_b3**

28.3 (22.2)


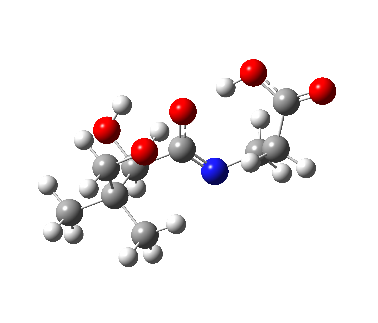

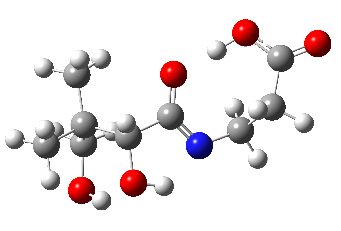

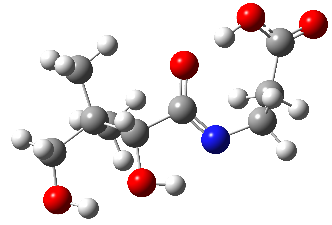

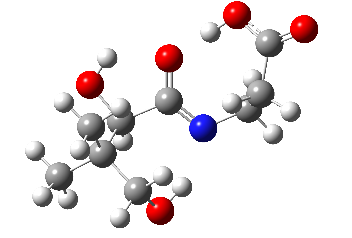

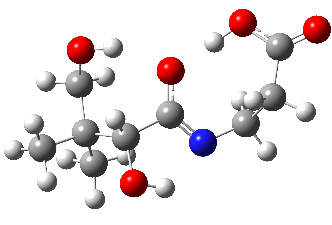

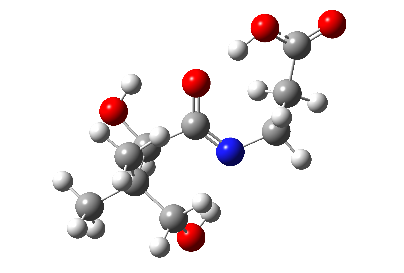


(h)

(i)

(k)

(j)

(l)

(m)

(n)

**Figure S6**. (h) IRMPD spectrum of [panto-H]^-^ together with (i-n) calculated spectra at the B3LYP/6-311++G(d,p) level. Spectra were scaled by a factor of 0.974. Relative free energies at 298 K are reported in kJ mol^-1^ at the B3LYP and MP2 (in parentheses) level.

**CaPa_a4**

31.8 (26.4)

**CaPa_a5**

25.5 (27.5)


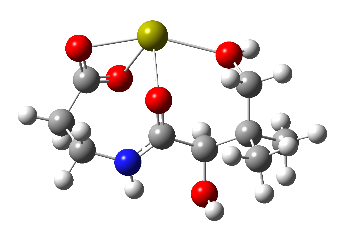

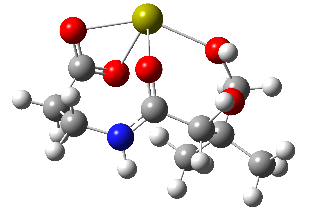


**CaPa_a3**

8.8 (15.7)

**CaPa_a2**

15.9 (6.1)


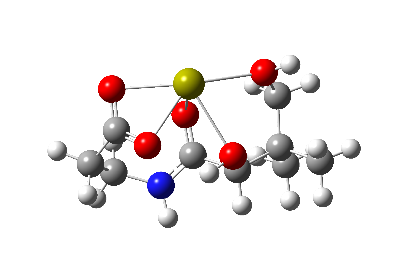

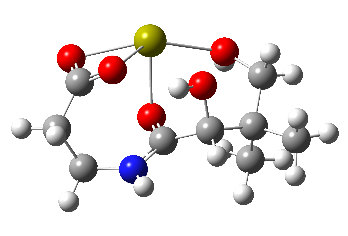


**CaPa_a1**

0.0 (0.0)

(f)

(e)

(d)

(c)


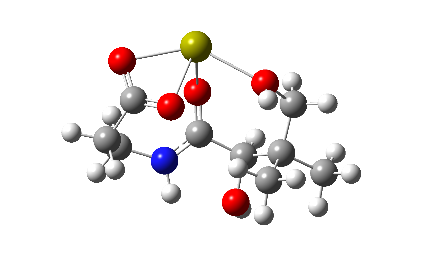


(b)

(a)

**Figure S7**. (a) IRMPD spectrum of [Ca(panto-H)]^+^ together with (b-f) calculated spectra at the B3LYP/6-311++G(d,p) level. Spectra were scaled by a factor of 0.974. Relative free energies at 298 K are reported in kJ mol^-1^ at the B3LYP and MP2 (in parentheses) level.

(l)

**CaPa_c1**

74.1 (58.8)

**CaPa_c4**

89.3 (72.7)

**CaPa_c3**

82.6 (69.6)


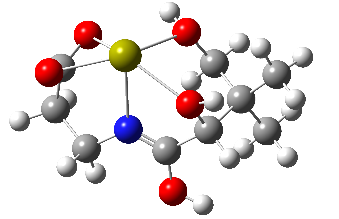


**CaPa_c2**

79.3 (62.0)


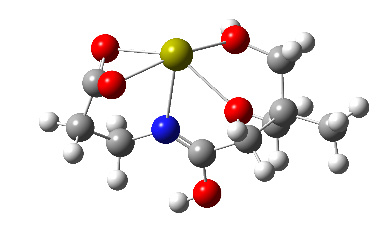

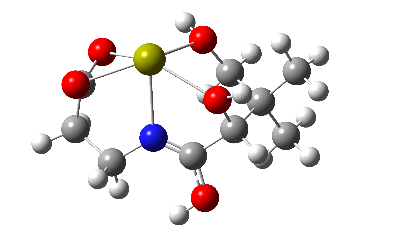

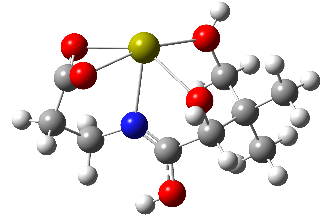


**CaPa_b1**

74.2 (79.6)


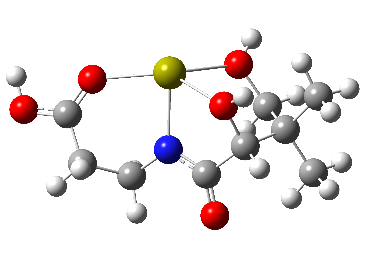


(k)

(j)

(i)

(h)

(g)

**Figure S8**. (g) IRMPD spectrum of [Ca(panto-H)]^+^ together with (h-l) calculated spectra at the B3LYP/6-311++G(d,p) level. Spectra were scaled by a factor of 0.974. Relative free energies at 298 K are reported in kJ mol^-1^ at the B3LYP and MP2 (in parentheses) level.

**CaPaW_a2**

15.4 (5.9)


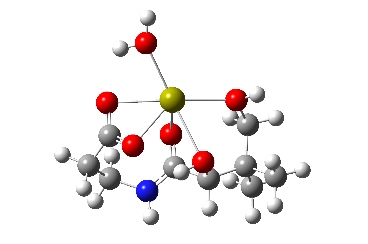


**CaPaW _a3**

11.1 (10.4)


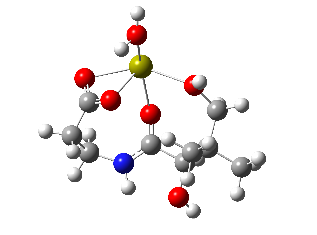


**CaPaW _a1**

0.0 (0.0)


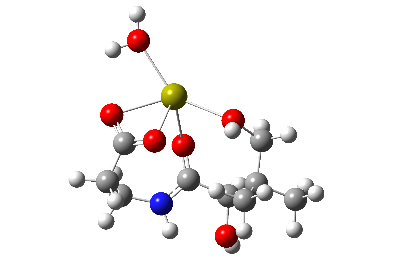


**CaPaW _a5**

18.8 (20.4)


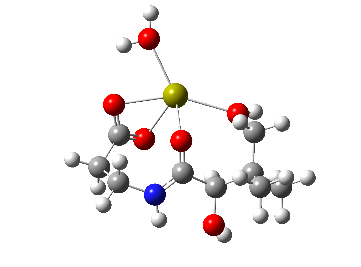

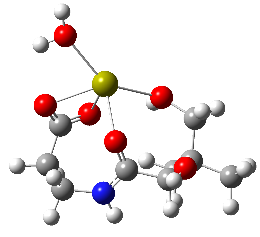


**CaPaW_a4**

15.6 (12.3)

(a)

(b)

(c)

(d)

(e)

(f)

(g)

**CaPaW _a6**

26.2 (20.7)


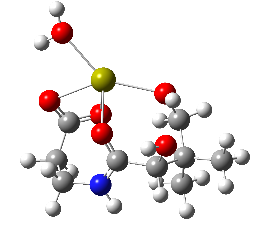


**Figure S9**. (a) IRMPD spectrum of [Ca(panto-H)(H_2_O)]^+^ together with (b-g) calculated spectra at the B3LYP/6-311++G(d,p) level. Spectra were scaled by a factor of 0.974. Relative free energies at 298 K are reported in kJ mol^-1^ at the B3LYP and MP2 (in parentheses) level.

**CaPaW_a7**

23.3 (25.8)


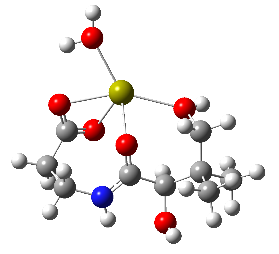


**CaPaW _a10**

68.6 (60.2)

**CaPaW _a8**

32.6 (41.8)


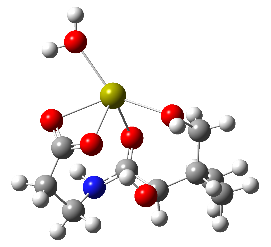


**CaPaW _a9**

53.5 (53.8)


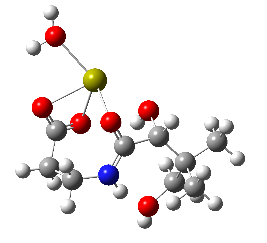

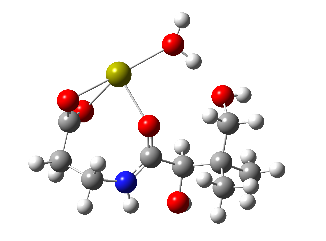


(h)

(i)

(j)

(k)

(l)

(m)

**CaPaW _c1**

80.5 (65.6)


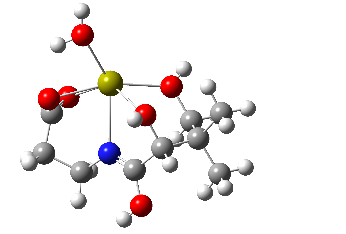


**Figure S10**. (h) IRMPD spectrum of [Ca(panto-H)(H_2_O)]^+^ together with (i-m) calculated spectra at the B3LYP/6-311++G(d,p) level. Spectra were scaled by a factor of 0.974. Relative free energies at 298 K are reported in kJ mol^-1^ at the B3LYP and MP2 (in parentheses) level.

(n)

(o)

(p)

(q)

(r)

(s)

**CaPaW _d1**

67.3 (67.7)


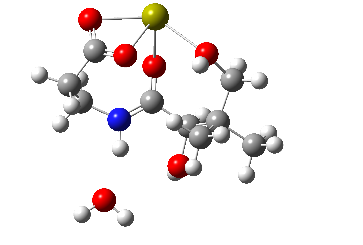


**CaPaW _a13**

85.5 (84.2)

**CaPaW _b1**

81.7 (81.2)

**CaPaW _a11**

79.9 (70.7)

**CaPaW _a12**

74.6 (72.1)


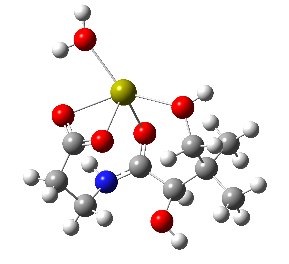

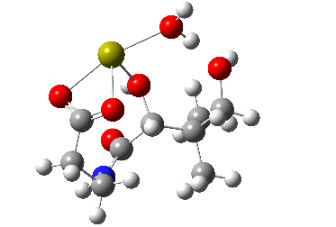

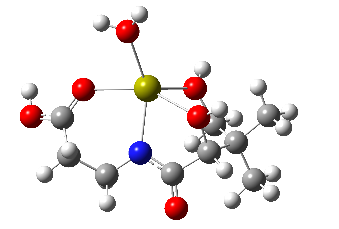

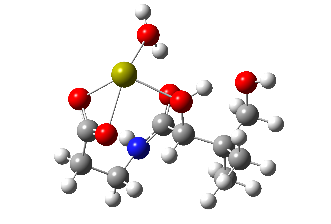


**Figure S11**. (n) IRMPD spectrum of [Ca(panto-H)(H_2_O)]^+^ together with (o-s) calculated spectra at the B3LYP/6-311++G(d,p) level. Spectra were scaled by a factor of 0.974. Relative free energies at 298 K are reported in kJ mol^-1^ at the B3LYP and MP2 (in parentheses) level.

**Table S1.** Thermodynamic data (kJ mol^-1^) for the most stable structures of [panto-H]^-^ calculated at either the B3LYP(-D3) or MP2 levels.

|  | B3LYP^a^ | | B3LYP-D3^a^ | | MP2//B3LYP^a,b^ | |
| --- | --- | --- | --- | --- | --- | --- |
|  | **H_rel_** | **G_rel_** | **H_rel_** | **H_rel_** | **H_rel_** | **G_rel_** |
| **Panto_a1** | 0.0 | 0.0 | 0.0 | 0.0 | 0.0 | 0.0 |
| **Panto_a2** | -0.6 | 0.9 | 0.7 | 0.2 | 0.0 | 1.5 |
| **Panto_a3** | 3.8 | 8.2 | -0.3 | 1.4 | 1.3 | 5.6 |
| **Panto_a4** | 17.8 | 17.9 | 18.4 | 15.9 | 16.2 | 16.4 |
| **Panto_a5** | 13.5 | 10.6 | 20.6 | 15.6 | 20.4 | 17.5 |
| **Panto_a6** | 18.2 | 19.3 | 17.9 | 17.1 | 17.0 | 18.2 |
| **Panto_b1** | 24.0 | 27.6 | 29.1 | 30.7 | 17.0 | 20.7 |
| **Panto_b2** | 25.0 | 28.5 | 28.6 | 30.4 | 17.6 | 21.1 |
| **Panto_b3** | 25.2 | 28.3 | 30.7 | 31.4 | 19.1 | 22.2 |
| **Panto_b4** | 27.6 | 29.7 | 33.7 | 33.3 | 23.3 | 25.4 |
| **Panto_b5** | 35.3 | 37.3 | 41.9 | 41.2 | 31.9 | 33.9 |
| **Panto_b6** | 42.3 | 45.7 | 44.6 | 46.1 | 32.4 | 35.8 |
| ^a^The 6-311++G(d,p) basis set was employed. ^b^Thermal corrections obtained from B3LYP calculations. | | | | | | |

**Table S2.** Observed IRMPD bands and calculated vibrational frequencies for the lowest lying structure **Panto_a1** and **Pato_b1** of deprotonated pantothenic acid, [panto-H]^-^.

|  | Calculated^a,b^ |  |  |
| --- | --- | --- | --- |
| IRMPD^a^ | **Panto_a1** | **Panto_b1** | Vibrational mode |
| 1001 | 1019 (42) |  | C2H_2_ rock + C3H_2_ rock |
| 1074 |  | 1034 (79) | C2H_2_ rock + C3H_2_ rock + C2'O2' stretch |
|  | 1055 (44) | 1050 (81) | C3H_2_ rock + C4'O' stretch |
| 1097 | 1081 (59) | 1072 (52) | C3N stretch + C2'O2' stretch |
| 1198 |  | 1166 (40) | C2H_2_ twist |
|  | 1187 (105) |  | NH bend + C2H_2_ wag + C3H_2_ twist |
| 1291 | 1288 (71) | 1327 (139) | C2H_2_ wag + C3H_2_ twist + O2'H bend |
| 1334 | 1318 (186) |  | C1C2 stretch + C3H bend + NH bend + sym carboxylate stretch |
| 1415 | 1405 (194) | 1400 (172) | O2'H bend + C2'H bend |
|  | 1421 (34) | 1472 (47) | O4'H bend + C4'H_2_ wag |
|  |  | 1483 (195) | O1bH bend |
| 1514 | 1516 (414) |  | NH bend |
| 1632 | 1627 (774) |  | antisym carboxylate stretch + NH bend |
|  | 1650 (229) | 1587 (326) | C1'O1' stretch + C1'N stretch |
|  |  | 1722 (540) | O1a stretch + O1bH bend |

^a^ In cm^-1^. ^b^ Intensities in parentheses in km mol^-1^. Vibrations with intensities lower than 30 km mol^-1^ are not reported.

**Table S3.** Thermodynamic data (kJ mol^-1^) for the most stable structures of [Ca(panto-H)]^+^ calculated at either the B3LYP(-D3) or MP2 levels.

|  | B3LYP^a^ | | B3LYP-D3^a^ | | MP2//B3LYP^a,b^ | |
| --- | --- | --- | --- | --- | --- | --- |
|  | **H_rel_** | **G_rel_** | **H_rel_** | **H_rel_** | **H_rel_** | **G_rel_** |
| **CaPa_a1** | 0.0 | 0.0 | 0.0 | 0.0 | 0.0 | 0.0 |
| **CaPa_a2** | 10.2 | 15.9 | 16.9 | 12.2 | 0.4 | 6.1 |
| **CaPa_a3** | 19.1 | 24.7 | 26.3 | 21.6 | 10.2 | 15.7 |
| **CaPa_a4** | 29.4 | 31.8 | 24.6 | 22.0 | 24.0 | 26.4 |
| **CaPa_a5** | 22.4 | 25.5 | 25.8 | 23.3 | 24.4 | 27.5 |
| **CaPa_c1** | 77.3 | 74.1 | 81.4 | 83.2 | 61.9 | 58.8 |
| **CaPa_c2** | 79.5 | 79.3 | 85.1 | 84.1 | 62.3 | 62.0 |
| **CaPa_c3** | 83.3 | 82.6 | 88.9 | 90.9 | 70.0 | 69.6 |
| **CaPa_c4** | 88.3 | 89.3 | 93.0 | 91.8 | 71.7 | 72.7 |
| **CaPa_b1** | 79.8 | 74.2 | 80.2 | 86.9 | 85.2 | 79.6 |
| ^a^The 6-311++G(d,p) basis set was employed. ^b^Thermal corrections obtained from B3LYP calculations. | | | | | | |

**Table S4.** Thermodynamic data (kJ mol^-1^) for the most stable structures of [Ca(panto-H)(H_2_O)]^+^ calculated at either the B3LYP(-D3) or MP2 levels.

|  | B3LYP^a^ | | B3LYP-D3^a^ | | MP2//B3LYP^a,b^ | |
| --- | --- | --- | --- | --- | --- | --- |
|  | **H_rel_** | **G_rel_** | **H_rel_** | **G_rel_** | **H_rel_** | **G_rel_** |
| **CaPaW_a1** | 0.0 | 0.0 | 0.0 | 0.0 | 0.0 | 0.0 |
| **CaPaW_a2** | 10.0 | 15.4 | 11.5 | 14.9 | 0.5 | 5.9 |
| **CaPaW_a3** | 8.8 | 11.1 | 7.6 | 9.4 | 8.1 | 10.4 |
| **CaPaW_a4** | 13.4 | 15.6 | 7.6 | 13.7 | 10.1 | 12.3 |
| **CaPaW_a5** | 16.0 | 18.8 | 17.4 | 18.4 | 17.7 | 20.4 |
| **CaPaW_a6** | 22.0 | 26.2 | 19.7 | 22.2 | 16.5 | 20.7 |
| **CaPaW_a7** | 20.3 | 23.3 | 21.3 | 22.6 | 22.8 | 25.8 |
| **CaPaW_a8** | 29.8 | 32.6 | 33.2 | 34.1 | 39.0 | 41.8 |
| **CaPaW_a9** | 52.1 | 53.5 | 55.4 | 54.6 | 52.5 | 53.8 |
| **CaPaW_a10** | 65.6 | 68.6 | 58.4 | 62.4 | 57.3 | 60.2 |
| **CaPaW_c1** | 81.0 | 80.5 | 85.7 | 84.3 | 66.1 | 65.6 |
| **CaPaW_d1** | 70.6 | 67.3 | 67.3 | 66.9 | 71.0 | 67.7 |
| **CaPaW_a11** | 75.4 | 79.9 | 68.6 | 72.5 | 66.1 | 70.7 |
| **CaPaW_a12** | 73.5 | 74.6 | 71.0 | 71.3 | 71.0 | 72.1 |
| **CaPaW_b1** | 92.8 | 81.7 | 99.2 | 89.5 | 92.3 | 81.2 |
| **CaPaW_a13** | 81.3 | 85.5 | 74.9 | 77.8 | 79.9 | 84.2 |
| ^a^The 6-311++G(d,p) basis set was employed. ^b^Thermal corrections obtained from B3LYP calculations. | | | | | | |

**Table S5.** Observed IRMPD bands and calculated vibrational frequencies for the lowest lying structures **CaPaW_a1** and **CaPaW_a2** of [Ca(panto-H)(H_2_O)]^+^.

|  | Calculated^a,b^ | |  |
| --- | --- | --- | --- |
| IRMPD^a^ | **CaPaW_1** | **CaPaW_2** | Vibrational mode |
| 972 | 958 (117) | 941 (74) | C4'O4' stretch + C2'O2' stretch + CH_3_ bend |
| 1020 |  | 993 (51) | C4'O4' stretch + CH_3_ bend |
|  |  | 1001 (32) | C1'C2' stretch + C3N stretch |
| 1085 | 1056 (63) |  | C2'O2' stretch + CH_2_ (all) rock |
| 1192 | 1177 (37) |  | O2'H bend + C2H_2_ twist |
| 1231 |  | 1231 (33) | O2'H bend + C2H_2_ twist |
| 1292 | 1272 (48) |  | NH bend + C2'H bend + C3H bend |
| 1379 |  | 1381 (35) | O2'H bend + C2'H bend + O4'H bend + C4'H bend |
|  | 1384 (33) |  | O4'H bend + C4'H bend + CH_3_ umbrella |
| 1450 | 1415 (86) | 1406 (114) | C2H_2_ scissor + C1C2 stretch + sym carboxylate stretch + CH_3_ umbrella |
|  | 1443 (92) | 1440 (46) | C2H_2_ scissor + C3H_2_ scissor |
| 1536 | 1513 (472) | 1520 (485) | asym carboxylate stretch + C2H_2_ twist + H_2_O scissor |
|  | 1548 (206) | 1527 (154) | NH bend (Amide II) |
| 1628 | 1585 (79) | 1579 (55) | H_2_O scissor |
|  | 1619 (383) | 1618 (309) | C1'O1' stretch (Amide I) |

^a^ in cm^-1^. ^b^ intensities in parentheses in km mol^-1^. Vibrations with intensities lower than 30 km mol^-1^ are not reported.
